# Quantum computing for transport network design problems

**DOI:** 10.1038/s41598-023-38787-2

**Published:** 2023-07-28

**Authors:** Vinayak V. Dixit, Chence Niu

**Affiliations:** School of Civil and Environmental Engineering, UNSW Sydney, Sydney, Australia

**Keywords:** Civil engineering, Engineering

## Abstract

Transport network design problem (TNDP) is a well-studied problem for planning and operations of transportation systems. They are widely used to determine links for capacity enhancement, link closures to schedule maintenance, identify new road or transit links and more generally network enhancements under resource constraints. As changes in network capacities result in a redistribution of demand on the network, resulting in changes in the congestion patterns, TNDP is generally modelled as a bi-level problem, which is known to be NP-hard. Meta-heuristic methods, such as Tabu Search Method are relied upon to solve these problems, which have been demonstrated to achieve near optimality in reasonable time. The advent of quantum computing has afforded an opportunity to solve these problems faster. We formulate the TNDP problem as a bi-level problem, with the upper level formulated as a Quadratic Unconstrained Binary Optimization (QUBO) problem that is solved using quantum annealing on a D-Wave quantum computer. We compare the results with Tabu Search. We find that quantum annealing provides significant computational benefit. The proposed solution has implications for networks across different contexts including communications, traffic, industrial operations, electricity, water, broader supply chains and epidemiology.

## Introduction

Transport network design problem (TNDP) has been a critical area for research and application, ranging from capacity upgradation, new transport links ranging from public transport to roads as well as scheduling, maintenance, and renewal programs. The feedback between demand and supply makes transport network design problems extremely hard and complex problems that are generally represented as a bilevel optimization problems^1^. These challenges have spurred significant innovation in this space with review articles synthesizing the progress every few years^2–6^.

A wide variety of solution methods have been explored ranging from time-consuming exact methods to metaheuristics that produce fast efficient solutions. However, recent advances in quantum computing has brought forth opportunities to provide a "*quantum leap*" in solving TNDP. We specifically use quantum annealing to solve the harder upper-level problem in the bi-level problem.

In this paper, we propose a new Quadratic unconstrained binary optimization (QUBO) model for the upper level. We also demonstrate the performance compared to existing state-of-the-art methods. The speed of the proposed models affords itself for real-time applications and fast if–then-else analysis.

The rest of the paper is organized as follows: Sect. “Literature review” summarizes the literature on quantum computing and optimization algorithms with a focus on TNDP; Sect. “Transportation network design problem” presents the mathematical formulation of this problem and the proposed solution algorithm; Sect. “Numerical results” describes the implementation of the performance of the proposed algorithm and Sect. “Conclusion” provides a discussion on the potential extensions of this research and synthesizes its contributions.

## Literature review

In this section, we summarize the current state of quantum computing particularly focusing on quantum annealing; though this section has been discussed in other papers, we find it appropriate to present it again for completeness. We also summarize the main contributions in TNDP to frame our contribution.

### Quantum computing

Research in quantum computing and algorithms over the past three decades has theoretically demonstrated the potential gains through "quantum speedup"^7^. At a fundamental level, quantum computers differ from classical computers in their ability to leverage quantum mechanical properties such as superposition, entanglement and interference to speed up computations.

There has been groundbreaking theoretical work that demonstrated quantum algorithms relying on quantum logic gates can provide significant speedups, one of the most celebrated being the Shor’s algorithm^8^, that demonstrated that quantum computers can solve the prime factorization problem exponentially faster than classical computers, having significant implications on cryptography. Recently "Quantum Supremacy" was demonstrated on a problem that would take a classical supercomputer 10,000 years to be completed by 53 qubit Sycamore processor in 200 seconds^9^. Applications of quantum algorithms in the field of transportation and traffic have been limited. Dixit and Jian^10^ used quantum gates for drive cycle analysis, which has applications to safety and emissions, as well as Dixit et al.^11^ solving the Scenario Based Stochastic Time Dependent Shortest Path.

Quantum computational engines based on quantum annealing are fundamentally different, for e.g. D-Wave quantum computers (https://www.dwavesys.com/). They rely on the process of "quantum annealing" to start from a particular system state to that of the final state defined by a Hamiltonian defining the feasible states. As is well known, finding minimum energy states in non-convex Hamiltonians is an NP-hard problem that classical computers take a long time to solve. A D-Wave's quantum annealer (QA) implements the optimization problem as a following time-dependent Ising Hamiltonian:

1 $$H_{{Q_{A} }} /h = \underbrace {{ - A\left( {\frac{t}{{t_{f} }}} \right)\sum\limits_{i} {\sigma _{i}^{X} } }}_{{Initial{\mkern 1mu} \,\,{\mkern 1mu} Hamiltonian}} + \underbrace {{B\left( {\frac{t}{{t_{f} }}} \right)\left( {\sum\limits_{i} {h_{i} } \sigma _{i}^{Z} + \sum\limits_{{i > j}} {J_{{ij}} \sigma _{i}^{Z} \sigma _{j}^{Z} } } \right)}}_{{Final\,\,{\mkern 1mu} {\mkern 1mu} Hamiltonian}},$$

where, ${t}_{f}$ is the annealing time, ${\sigma }_{i}^{X}$ and ${\sigma }_{i}^{Z}$ are the Pauli matrices acting on qubit i, and ${h}_{i}$ and ${J}_{ij}$ are the qubit biases and coupling strengths, respectively.

The operating temperature of quantum annealing is less than 15 millikelvin. The ground state of the $n$-qubit system is relaxed to the uniform superposition of all computational basis states $|\psi_{0} \left( 0 \right) = \left[ {\left( {|\left. { + 1} \right\rangle + |\left. { - 1} \right\rangle } \right) \otimes \cdots \otimes \left( {|\left. { + 1} \right\rangle + |\left. { - 1} \right\rangle } \right)} \right]/\sqrt {2^{n} }$. During the annealing process, each qubit station in $|{\psi }_{0}\left({t}_{a}\right)\rangle$ (${t}_{a}$ is the time point of the end of the annealing) can be determined as the lowest energy solution of the Ising Hamiltonian^12^:

2 $${H}_{Ising}\left(s\right)=\sum_{i}{h}_{i}{s}_{i}+\sum_{i>j}{J}_{ij}{s}_{i}{s}_{j},$$

where ${s}_{i}=\pm 1$ are Ising spin variables. Following a preset annealing schedule given by the time-dependent functions $A\left(t/{t}_{f}\right)$ and $B\left(t/{t}_{f}\right)$, the Hamiltonian of the system slowly changes from the initial to the final Hamiltonian state, which encodes the solution of the given optimization problem.

The Quantum Annealer solves Ising minimization problems, which are isomorphic to a Quadratic Unconstrained Binary Optimization (QUBO) Problem that are NP-Hard problems of the form:

3 $$\mathrm{Obj }:={x}^{T}Qx,$$

where *x* is a vector of N binary variables and Q is an NxN matrix representing^13^ the coefficients of the quadratic terms. The diagonal terms of Q are mapped to ${h}_{i}$ and the cross terms are mapped to ${J}_{ij}$ in the final Hamiltonian.

The computational benefits afforded by Quantum annealers have led to a significant foray into representing some of the transportation problems as a QUBO problem that could be solved on a D-Wave. These include (a) Travelling Salesman Problem that has been thoroughly reviewed and evaluated by Warren^14^, (b) Travelling Salesman Problem with Time Windows^15^, (c) Vehicle Routing Problems as well as its variants such as multi-depot capacitated vehicle routing problem (MDCVRP) and its dynamic version^16^, (d) Traffic signal control^17^, and (e) Redistributing and rerouting vehicles for optimal network utilization^18^. It is important to note that Quantum annealing is a meta-heuristic^19^; though it has repeatedly been demonstrated to outperform classical computers to get to efficient solutions quicker, they do not guarantee optimality until exhausting the search space.

### Transportation network design problems

TNDP has been explored in various concepts ranging from decisions on improvements in road capacity^20,21^, optimal facilities location^22^ and long-term optimal decisions related to the infrastructure for transportation networks^23^. In this particular work, we focus on the problem of identification of optimal capacity investment under resource constraints, in our case it is the number of links that can be improved, under deterministic travel demand. The method proposed for this problem can be easily adapted to other TNDP problems.

TNDP is formulated as a Bi-level programming problem, which is also referred to as a leader–follower problem. Decision-makers’ or leaders' problem corresponds to the upper level, with leaders designing and planning the transport network. The traveler's or follower's problem is referred to as the lower-level problem, where the travellers react to the planning decisions by the leader to re-assign themselves to choose their optimal modes and routes. The problem is mathematically formulated as:

4 $$[{\mathrm{U}}_{0}]\underset{u}{\mathrm{min}}F\left(u,v\left(u\right)\right),$$

5 $$s.t. G\left(u,v(u)\right)\le 0,$$

where $v(u)$ is determined by the lower-level problem

6 $$\left[{\mathrm{L}}_{0}\right]\underset{v}{\mathrm{min}}f\left(u,v\right),$$

7 $$s.t. g\left(u,v\right)\le 0.$$

In the upper-level problem, $u$ is a vector of decision variables, $F$ represents the objective function, and $G$ is a vector function of constraints for the upper-level problem. In the lower-level problem, $f$ denotes the objective function and $g$ is the vector function of constraint. $v(u)$ is the response function, capturing the user reaction on traffic assignment for specific network design decisions. Therefore, $v(u)$ is an optimal solution of L0. TNDP is commonly modelled with the upper level being formulated as minimizing the total cost objective function and the lower level determining the link flows under the user equilibrium (UE) assumption.

A recent review paper by Jia et al.^6^ presents the most current and comprehensive review for this area, which found heuristic algorithms such as Branch and bound, GA and Tabu search methods being predominantly used to solve TNDP. Cantarella et al.^24^ used different metaheuristic algorithms such as Hill Climbing, Simulated Annealing, Tabu Search, GA and Path Relinking to solve the TNDP with considering the network topology and link capacity. Their results indicated that Tabu Search performed better than other algorithms in terms of optimal solutions and computation times. The tabu search is also a well-used metaheuristic framework with high computational efficiency and solution quality for the transportation optimization problems such as vehicle routing problems and bus assignment problems^25–29^. Therefore, we benchmark the performance of quantum computing with Tabu Search. Specifically, the MST2 multistart tabu search algorithm is used to solve the quadratic unconstrained binary optimization (QUBO) problem with a dimod sampler^26,30^.

In this paper, we focus on the differences in optimal solutions and computation times between quantum computing and traditional meta-heuristics (Tabu Search algorithm) to solve the TNDP for different road network sizes. To deploy quantum computing to solve the TNDP, We formulate the problem as a QUBO problem, which makes a quantum annealing-based quantum computing method possible.

## Transportation network design problem

In this section, we present the mathematical formulation of the TNDP and the proposed solution algorithm.

### Problem formulation

We use graph theory to represent mathematically this problem and the notation used throughout the paper is summarized in Table 1.

**Table 1:** Mathematical notation.

$V$ Set of nodes
$A$ Set of links, |A| is the number of links
$W$ Set of OD pairs
$K$ Set of paths
${x}_{a}$ Traffic flow on link $a$
${t}_{a}$ Travel time on link $a$
${t}_{{f}_{a}}$ Free flow travel time on link $a$
${c}_{a}$ Capacity per lane of link $a$
${l}_{a}$ Lane number of link $a$
$\alpha$, $\beta$ Attributes of the travel time function
${y}_{a}$ Decision binary variable denoting whether link $a$ is expended
${\Delta }_{a}$ The extend of expansion of link $a$ capacity
$N$: Maximum number of links to be improved, i.e. Budget
${f}_{k}^{rs}$ Traffic flow on path $k$ connecting O-D pair $r-s$;${\mathrm{f}}^{\mathrm{rs}}=\left(\dots ,{f}_{k}^{rs},\dots \right)$
${q}_{rs}$ Demand between origin $r$ and destination $s$
${s}_{i}$ Slack variables for the budget constraint $i\in (1\dots \left|A\right|)$
$${\delta }_{a,k}^{rs}$$ Indicator variables

As discussed earlier, the problem is formulated as a bilevel program with the upper level being a total system travel time minimization problem with budget constraints on the number of links where capacity improvements can be made, with the lower level being a traffic assignment based on User Equilibrium. An improvement in link capacity would lead to a redistribution of traffic flow on the road network based on UE, thus affecting traffic congestion and the TSTT.

The upper-level problem represents the total system travel time as an objective function with a budget constraint:

8 $$\mathrm{min}{\sum }_{a}{x}_{a}{t}_{a}({x}_{a},{y}_{a}),$$

9 $${\sum }_{a}{y}_{a}\le N.$$

The lower-level problem is the standard user equilibrium:

10 $$\mathrm{min}z\left(\mathbf{x}\right)=\sum_{a\in A}{\int }_{0}^{{x}_{a}}{t}_{a}(\omega )d\omega .$$

Subject to

11 $$\sum_{k\in K}{f}_{k}^{rs}={q}_{rs} \forall r,s\in W$$

12 $${f}_{k}^{rs}\ge 0 \forall k\in K$$

13 $${x}_{a}=\sum_{r}\sum_{s}\sum_{k}{f}_{k}^{rs}{\delta }_{a,k}^{rs}\,\, \forall \,\,a\in A$$

The link travel time function is represented by the traditional Bureau of Public Roads (BPR) link cost function.

14 $${t}_{a}\left({x}_{a}\right)={t}_{{f}_{a}}\left(1+\alpha {\left(\frac{{x}_{a}}{{c}_{a}}\right)}^{\beta }\right),$$

With the capacity of link $a$ expressed as a function of the decision variable ${y}_{a}$, i.e. whether the link is chosen for capacity expansion or not is written as:

15 $${c}_{a}=\left({l}_{a}+{\Delta }_{a}{y}_{a}\right).$$

Therefore, the link travel time as a function ${x}_{a}$ and ${y}_{a}$ can be written as:

16 $${t}_{a}\left({x}_{a}, {y}_{a}\right)={t}_{{f}_{a}}{y}_{a}\left(1+\alpha {\left(\frac{{x}_{a}}{{c}_{a}\left({l}_{a}+{\Delta }_{a}\right)}\right)}^{\beta }\right)+{t}_{{f}_{a}}\left(1-{y}_{a}\right)\left(1+\alpha {\left(\frac{{x}_{a}}{{c}_{a}\left({l}_{a}\right)}\right)}^{\beta }\right),$$

17 $${t}_{a}\left({x}_{a}, {y}_{a}\right)={t}_{{f}_{a}}\left(1+\alpha {{\left(\frac{{x}_{a}}{{c}_{a}\left({l}_{a}\right)}\right)}^{\beta }+\alpha y}_{a}\left[{\left(\frac{{x}_{a}}{{c}_{a}\left({l}_{a}+{\Delta }_{a}\right)}\right)}^{\beta }-{\left(\frac{{x}_{a}}{{c}_{a}{l}_{a}}\right)}^{\beta }\right]\right),$$

This representation of the travel time function is a critical transformation that enables a QUBO formulation that then enables the use of quantum annealing methods. Appendix A provides a way to generalize the formulation to include an additional choice of link improvements, both from a free flow speed perspective or capacity, as well as a more general budget constraint. We use the simpler TNDP problem to evaluate the computational experience.

### QUBO formulation

The upper-level problem, which can be represented as a Quadratic Constrained Optimization Problem formulation using the Lagrangian can be converted into a Quadratic Unconstrained Optimization (QUBO) problem.

The objective function of the upper level shown in Eq. (7) in conjunction with the travel time function shown in Eq. (16), can be written as Eq. (17). As can be observed in Eq. (17), Term 1 is a constant Total System Travel Time, w.r.t. the upper-level problem, determined from the lower-level problem. Term 2, has the decision variables. The upper-level objective function is:

18 $$\min \underbrace {{\sum _{a} \left( {x_{a} t_{{f_{a} }} \left( {1 + \left( {\frac{{x_{a} }}{{c_{a} l_{a} }}} \right)^{\beta } } \right)} \right)}}_{{{\text{Term}}{\mkern 1mu} {\mkern 1mu} 1}} + \underbrace {{\sum _{a} \left( {\alpha x_{a} t_{{f_{a} }} \left[ {\left( {\frac{{x_{a} }}{{c_{a} \left( {l_{a} + \Delta _{a} } \right)}}} \right)^{\beta } - \left( {\frac{{x_{a} }}{{c_{a} l_{a} }}} \right)^{\beta } } \right]y_{a} } \right)}}_{{{\text{Term}}{\mkern 1mu} {\mkern 1mu} 2}}.$$

The budget constraint in the upper-level is an inequality, which can be easily converted to an equality (Eq. 19) through an addition of binary (0 or 1) slack variables ${s}_{i}$, where *i* ranges from 1…|A|.

19 $${\sum }_{a}{y}_{a}+{\sum }_{i=1..|A|}{s}_{i}=N.$$

Taking the equivalent Lagrangian for the constrained optimization problem, i.e. Equations (18) and (19), we generate Eq. (20), where $\lambda$ is the corresponding Lagrange multiplier for Eq. (19).

20 $$\mathrm{min}{\sum }_{a}{x}_{a}{t}_{a}({y}_{a})+\lambda {\left({\sum }_{a}{y}_{a}+{\sum }_{i=1..N}{s}_{i}-N\right)}^{2}.$$

Equation (20) can be algebraically reduced to a QUBO by recognizing that the square of a binary variable is the binary variable itself. This is shown in Eq. (21) below.

21 $$\mathrm{min}{\sum }_{a}\left({x}_{a}{t}_{{f}_{a}}\left(1+{\left(\frac{{x}_{a}}{{c}_{a}{l}_{a}}\right)}^{\beta }\right)\right)+{\sum }_{a}\left(\alpha {x}_{a}{t}_{{f}_{a}}\left[{\left(\frac{{x}_{a}}{{c}_{a}\left({l}_{a}+{\Delta }_{a}\right)}\right)}^{\beta }-{\left(\frac{{x}_{a}}{{c}_{a}{l}_{a}}\right)}^{\beta }\right]{y}_{a}\right)+\lambda \left(\left(1-2N\right){\sum }_{a}{y}_{a}+{\sum }_{i=1..N}\left(1-2N\right){s}_{i}+{\sum }_{i\in A}{\sum }_{j\in A s.t i<j}2{y}_{i}{y}_{j}+{\sum }_{i=1..|A|}{\sum }_{a}2{s}_{i}{y}_{a}+{\sum }_{i=1..|A|}{\sum }_{j=i+1..|A| s.t i<j}2{s}_{i}{s}_{j}+{N}^{2}\right).$$

Equation (21) presents the QUBO formulation that can be implemented on a quantum annealing computer. However, we still need to determine reasonable Lagrange multipliers, which will be discussed in the following section.

### QUBO solution method

The TNDP is solved by iteratively solving the lower-level problem and the upper-level QUBO problem. We implement the QUBO on a D-Wave Advantage quantum computer. The D-Wave’s Quantum Processing Unit (QPU) with only 5436 qubits is connected based on the Pegasus topology. However, several qubits are not connected. The limited number of qubits and connectivity creates significant challenges to embed and solve large problems. The D-Wave system attempts to navigate the connectivity issue by copying an optimization variable to multiple qubits, which is also referred to as *chain strength*, which not only reduces the number of qubits, but also errors in one qubit propagates significantly to affect the quality of the solution.

The upper diagonal matrix containing the coefficients of the QUBO is provided as an input to the D-Wave embedding system, as part of efficiently embedding the problem onto the chip, the system scales the coefficients to be between $-1$ and $1$. This makes the choice of the Lagrange multipliers critical. Having extremely large multipliers would scale down the coefficient values close to zero, which would make it hard to distinguish them from Noise. Therefore, we need to choose a large Lagrange multiplier that is in the range of the coefficients.

**Figure 1:**
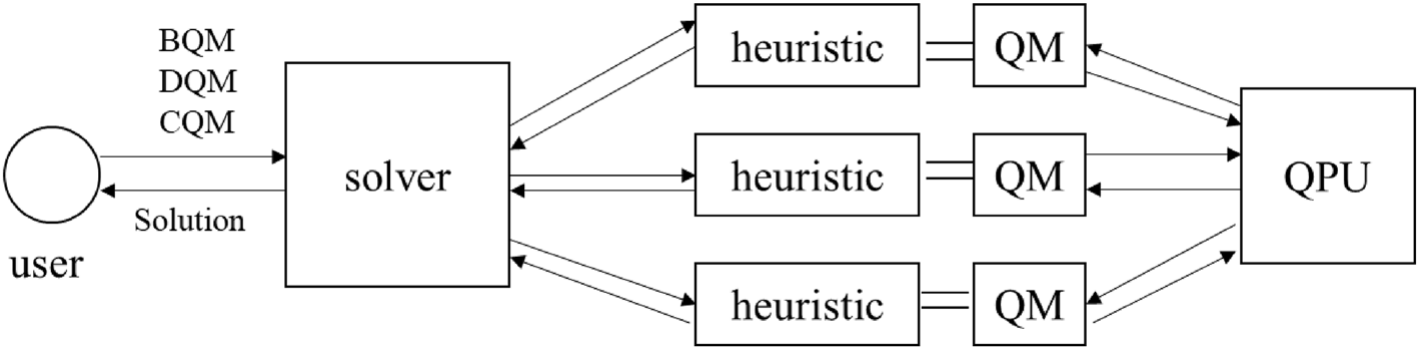
Structure of a hybrid solver in hybrid solver service^31^.

Given that the TNDP has been formulated as a QUBO problem for the first time, we explore the computational experience and trends by using D-Wave's pure quantum computer, and the Hybrid solver for larger problems with the performance of a Win64 i7 machine, 2.9Ghz and 8 Gb of RAM. For the Hybrid solver, as shown in Fig. 1, the problem is computed by a number of heuristic solvers to search for good-quality solutions by state-of-the-art CPU and/or GPU parallelly. Then the quantum modules (QM) formulate and send quantum queries to a D-Wave QPU which guides the heuristic search and improves the solution quality. The D-Wave Hybrid Binary Quadratic Model Version 2 is utilized in this study for larger binary problems.

## Numerical results

In this section, we evaluate the performance of using D-Wave’s quantum computer on benchmark networks and compare the computational experiences.

### Benchmark networks

The benchmark networks used are shown in Table 2, with their characteristics. The networks are available at https://github.com/bstabler/TransportationNetworks.

**Table 2:** Network characteristics.

Network	No. of nodes	No. of links	No. of trips
Sioux falls	24	76	20,000.0
EMA	74	258	65,576.38
Friedrichshain-center	224	523	11,205.10
Berlin-Prenzlauerberg-center	352	749	16,659.92
Berlin-Tiergarten	361	766	10,754.87
Berlin-Mitte-center	398	871	11,481.92
Anaheim	416	914	104,694.40
Winnipeg-Asym	1057	2535	1.36e + 006
Terrassa-Asym	1609	3264	2.52e + 007
Hessen-Asym	4660	6674	7.13e + 007

In D-Wave quantum computing, the annealing schedule was defined by a series of pairs of floating-point numbers that identify points in the schedule where the slope was changed. For each pair of numbers, the first element represented the time $t$ in microseconds, and the second element represented the normalized persistent current $s$, which rangeed from 0 to 1. The resulting schedule was a piecewise-linear curve formed by connecting the provided points^13^. In our experiments, the default setting ($[\left[0, 1\right], \left[0.5, 0.5\right], [1, 1]]$) was used. In addition, the adjustment of annealing schedule was not available in the Leap’s hybrid solver, where only the default setting was used. The Lagrange multipliers was set based on the best estimate of the objective function’s value, i.e. TSTT and the running time was set based on the QPU access time estimation method in Ocean software^32^.

As mentioned in Sect. “QUBO solution method”, due to limits with qubits, the pure quantum annealing approach could only be evaluated on a network with a small number of links, i.e. Sioux Falls, see Table 3. The Pure method had a similar performance to Tabu Search with respect to the quality of the optimal solution, however, the pure quantum annealing was quicker taking between 0.20 and 0.22 s, as compared to Tabu Search, which took between 0.30 and 0.4 s.

**Table 3:** Computational results of small network design problems.

Network	No. of nodes	No. of links	Budget percentage	Computational time (s)		ΔObj. %
				Tabu search	Pure	
Sioux	24	76	10%	0.33	0.22	0.08%
			20%	0.40	0.22	0.00%
			30%	0.30	0.21	0.00%
			40%	0.34	0.21	0.00%
			50%	0.30	0.20	0.00%

ΔObj. % = (TSTT_Pure_-TSTT_Tabu_)/TSTT_Pure_.

**Figure 2:**
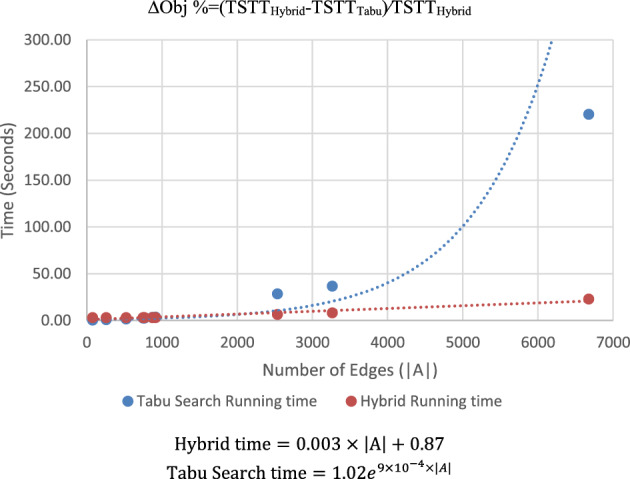
Computational experience of benchmark networks.

On larger networks we use the hybrid algorithm. The computational results for benchmark networks TNDP which are solved using Tabu Search and Hybrid solvers are given in Table 4. For the domain of problems tested, when the number of links is small (roughly less than 500), the Tabu Search method is faster in terms of absolute run times. However, the CPU run times increase rapidly as shown in Fig. 2. This is expected given the NP-hardness of integer programming. However, D-Wave’s hybrid method using the quantum annealer provides significant computational benefit, with a maximum was 11 times faster. The computational experience was found to be linear for the domain of problems tested in this paper. Therefore, the order of improvement in computation experience improved as the size of the network increased.

**Table 4:** Computational results of benchmark network design problems.

Network	No. of nodes	No. of links	Budget percentage	Computational time (s)		ΔObj. %
				Tabu search	Hybrid	
Sioux	24	76	10%	0.33	2.98	0.00%
			20%	0.40	2.99	0.00%
			30%	0.30	2.99	0.00%
			40%	0.34	3.00	0.00%
			50%	0.30	2.99	0.00%
EMA	74	258	10%	0.73	3.00	0.00%
			20%	0.72	2.99	0.00%
			30%	0.72	3.00	0.01%
			40%	0.72	2.98	0.01%
			50%	0.69	3.00	0.01%
Friedrichshain-center	224	523	10%	1.48	3.00	0.00%
			20%	1.60	2.99	0.00%
			30%	1.63	2.99	0.00%
			40%	8.74	3.00	0.00%
			50%	2.59	2.99	0.00%
Berlin-prenzlauerberg-center	352	749	10%	2.40	2.99	0.00%
			20%	2.70	2.99	0.00%
			30%	3.67	2.99	0.00%
			40%	3.24	2.99	0.00%
			50%	3.28	3.00	0.00%
Berlin-tiergarten	361	766	10%	2.53	2.99	0.00%
			20%	2.86	2.99	0.00%
			30%	3.25	3.00	0.00%
			40%	4.11	2.99	0.00%
			50%	3.02	2.99	0.00%
Berlin-mitte-center	398	871	10%	3.12	2.99	0.00%
			20%	3.53	3.00	0.00%
			30%	4.12	3.01	0.00%
			40%	71.89	2.99	0.00%
			50%	5.24	2.99	0.00%
Anaheim	416	914	10%	3.35	3.00	0.00%
			20%	3.01	2.99	0.00%
			30%	3.45	3.00	0.00%
			40%	3.49	2.98	0.00%
			50%	4.13	2.99	0.00%
Winnipeg-Asym	1057	2535	10%	28.36	6.35	0.00%
			20%	30.46	6.44	− 0.22%
			30%	40.83	6.44	− 0.11%
			40%	42.17	6.44	0.01%
			50%	40.44	6.44	− 0.08%
Terrassa-Asym	1609	3264	10%	36.72	8.10	0.00%
			20%	41.03	8.10	0.08%
			30%	37.56	8.10	− 0.19%
			40%	40.11	8.09	0.34%
			50%	46.29	8.08	0.08%
Hessen-Asym	4660	6674	10%	220.40	22.80	0.00%
			20%	266.19	23.00	– 2.01%
			30%	213.72	22.94	− 0.30%
			40%	205.92	23.06	− 2.03%
			50%	217.86	23.03	− 1.50%

ΔObj % = (TSTT_Hybrid_-TSTT_Tabu_)⁄TSTT_Hybrid_.

Though the quality of the optimal solution delivered by the Hybrid and Tabu Search were similar for small problems, the hybrid approach provided a better quality of optimal solution for larger problems (See Hessen-Asym in Table 4), with some solutions being almost 2% better.

## Conclusion

In this paper, we are the first to develop a novel QUBO formulation and apply quantum computing to the Transport Network Design Problem. We implement the solution method for TNDP using the D-Wave quantum computer. We evaluate the performance of quantum computing with the state-of-the-art Tabu Search Method on benchmark networks.

For small networks (the number of links is less than 150), the TNDP was able to be solved directly on the D-Wave QPU, with its computational time being smaller than Tabu Search. However, due to limitations with larger qubits with larger networks, we need to employ D-Wave’s hybrid quantum computing method. Based on the novel TNDP formulation as a QUBO, that affords using an Ising based quantum annealer, we empirically found that the quantum hybrid algorithm provides significant computational benefit. With regards to the size of the networks tested, we have tested and evaluated it on real-world large networks with almost 6674 links. Given that we utilize a hybrid quantum approach, conclusions regarding the possibility of the Ising model putting TNDP in BQP would be incorrect.

This study demonstrates the great potential of quantum computing in solving large-scale transportation problems. It is worth noting that with more qubits, better connectivity and error correction, we can see faster and more reliable QPU in the near future, that can solve these problems orders of magnitude faster.

## Supplementary Information

Supplementary Information.

## Data Availability

The data that support the findings of this study are available from the corresponding author upon reasonable request.
