# Supplementary material for: Quantum computing for transport network design problems
Source: Sci Rep. 2023 Jul 28;13:12267. doi: 10.1038/s41598-023-38787-2 (PMC10382597; doi:10.1038/s41598-023-38787-2)
Supplement: Supplementary file 1 — Supplementary Information. [file 41598_2023_38787_MOESM1_ESM.pdf]

Appendix A

It is important to note that in the current representation of travel time in Equation 16 provides only one possibility to increase capacity on a link. However, this can be easily expanded to additional improvements ranging from free flow travel time ($t^j_{f_a}$) and capacity (Δ^j_a). This is shown in Equation A1. Using Equation A1, the objective function of the upper level can be written as Equation A2.

$$t_a(x_a, Y_a) = t_{f_a}\left(1+\alpha\left(\frac{x_a}{c_a(l_a)}\right)^{\beta}\right) + \sum_{j\in J_a}\left[t^j_{f_a}y^j_a\left(1+\alpha\left(\frac{x_a}{c_a\left(l_a+\Delta^j_a\right)}\right)^{\beta}\right) - t_{f_a}y^j_a\left(1+\alpha\left(\frac{x_a}{c_a(l_a)}\right)^{\beta}\right)\right] \quad \text{(A1)}$$

$$\min \sum_a\left(x_a t_{f_a}\left(1+\left(\frac{x_a}{c_a l_a}\right)^{\beta}\right)\right) + \sum_a\sum_{j\in J_a}\left[t^j_{f_a}y^j_a\left(1+\alpha\left(\frac{x_a}{c_a\left(l_a+\Delta^j_a\right)}\right)^{\beta}\right) - t_{f_a}y^j_a\left(1+\alpha\left(\frac{x_a}{c_a(l_a)}\right)^{\beta}\right)\right] \quad \text{(A2)}$$

The budget constraint (Equation 8) in the upper-level is an inequality only associated to the number of links. However, this can be generalized to a monetary budget constraint (an integer $\lfloor B\rfloor$), with corresponding cost of link improvement being denoted by w^j_a, shown in Equation A3. Using slack variables this can be converted to an equality constraint, as shown in Equation A4.

$$\sum_a\sum_{j\in J_a} w^j_a y^j_a \le \lfloor B\rfloor \quad \text{(A3)}$$

$$\sum_a\sum_{j\in J_a} w^j_a y^j_a + \sum_{i=1\ldots\lfloor B\rfloor} s_i = \lfloor B\rfloor \quad \text{(A4)}$$

Using the Lagrangian form for the upper level problem and using Equation A2 and A4, we get Equation A5.

$$\min \sum_a\left(x_a t_{f_a}\left(1+\left(\frac{x_a}{c_a l_a}\right)^{\beta}\right)\right) + \sum_a\sum_{j\in J_a}\left[t^j_{f_a}y^j_a\left(1+\alpha\left(\frac{x_a}{c_a(l_a+\Delta_a)}\right)^{\beta}\right) - t_{f_a}y^j_a\left(1+\alpha\left(\frac{x_a}{c_a(l_a)}\right)^{\beta}\right)\right] + \lambda\left(\sum_a\sum_{j\in J_a} w^j_a y^j_a + \sum_{i=1\ldots\lfloor B\rfloor} s_i - \lfloor B\rfloor\right)^2 \quad \text{(A5)}$$

Equation A5 can be algebraically reduced to a QUBO by recognizing that the square of a binary variable is the binary variable itself. This is shown in Equation A6 below.

$$
\begin{aligned}
\min \sum\nolimits_a & \left(x_a t_{f_a} \left(1 + \left(\frac{x_a}{c_a l_a} \right)^{\beta} \right) \right) \\
& + \sum\nolimits_a \sum_{j \in J_a} \left[t_{f_a}^j y_a^j \left(1 + \alpha \left(\frac{x_a}{c_a (l_a + \Delta_a)} \right)^{\beta} \right) \right. \\
& \left. - t_{f_a} y_a^j \left(1 + \alpha \left(\frac{x_a}{c_a (l_a)} \right)^{\beta} \right) \right] \\
& + \lambda \left(\left(w_a^j - 2\lfloor B \rfloor \right) w_a^j \sum\nolimits_a \sum\nolimits_{j \in J_a} y_a^j + \sum\nolimits_{i=1..\lfloor B \rfloor} (1 - 2\lfloor B \rfloor) s_i \right. \\
& + \sum\nolimits_{a \in A, i \in J_a} \sum\nolimits_{a' \in A, j \in J_{a'}} 2 y_a^i y_{a'}^j + \sum\nolimits_{i=1..\lfloor B \rfloor} \sum\nolimits_{a \in A, j \in J_a} 2 s_i y_a^j \\
& \left. + \sum\nolimits_{i=1..\lfloor B \rfloor} \sum\nolimits_{j=i+1..\lfloor B \rfloor \; s.t \; i<j} 2 s_i s_j + \lfloor B \rfloor^2 \right)
\end{aligned}
\tag{A6}
$$

This formulation can be used for more general Transport Network Design Problems.
